# Population Pharmacokinetics and Dosing Optimization of Vancomycin in Pediatric Liver Transplant Recipients

**DOI:** 10.1128/Spectrum.00460-21

**Published:** 2021-10-06

**Authors:** Kensuke Shoji, Jumpei Saito, Hidenori Nakagawa, Takanori Funaki, Akinari Fukuda, Seisuke Sakamoto, Mureo Kasahara, Jeremiah D. Momper, Edmund V. Capparelli, Isao Miyairi

**Affiliations:** a Division of Infectious Diseases, Department of Medical Subspecialties, National Center for Child Health and Development, Tokyo, Japan; b Department of Pharmacy, National Center for Child Health and Development, Tokyo, Japan; c Organ Transplantation Center, National Center for Child Health and Development, Tokyo, Japan; d Skaggs School of Pharmacy and Pharmaceutical Sciences, University of California, San Diego, La Jolla, California, USA; e Division of Host-Microbe Systems and Therapeutics, University of California, San Diego, La Jolla, California, USA; f Department of Microbiology, Immunology, and Biochemistry, University of Tennessee Health Science Center, Memphis, Tennessee, USA; University of Calgary

**Keywords:** vancomycin, children, liver transplantation, population pharmacokinetics

## Abstract

Methicillin-resistant Staphylococcus aureus infections are a significant cause of morbidity and mortality in pediatric liver transplant (LT) recipients. Physiological changes following LT may affect vancomycin pharmacokinetics; however, appropriate dosing to achieve sufficient drug exposure (i.e., 24-h area under the concentration-time curve [AUC_24_]/MIC ≥ 400) in pediatric LT recipients has not been reported. This retrospective pharmacokinetics study of LT recipients aged <18 years utilized data on patient characteristics with vancomycin concentrations and dosing information obtained from electronic medical records. Population pharmacokinetics analysis was conducted by nonlinear mixed-effects modeling with the Phoenix NLME software. Potential covariates were screened with univariate and multivariate analysis. Monte Carlo simulations were performed using the final model to explore appropriate dosing. The study included 270 pharmacokinetics profiles encompassing 1,158 concentrations measured in 161 patients. The median age was 13.3 (interquartile range, 7.6 to 53.5) months, serum creatinine (sCr) was 0.16 (0.12 to 0.23) mg/dl, and days from LT (DFLT) was 17 (6 to 31). Multivariate analysis demonstrated that lower sCr and shorter DFLT were associated with higher clearance. By *post hoc* estimation, the average clearance and volume of distribution were 0.18 liters/h/kg and 1.01 liters/kg, respectively. The Monte Carlo simulations revealed that only 16% of patients achieved an AUC_24_/MIC of ≥400 with the assumed vancomycin MIC of 1 μg/ml. DFLT and sCr were significant covariates for vancomycin clearance in pediatric LT recipients. Standard vancomycin dosing may be insufficient, and higher or more frequent dosing may be required to achieve an AUC_24_/MIC of ≥400 in pediatric LT recipients with normal renal function.

**IMPORTANCE** We evaluated vancomycin pharmacokinetics in pediatric LT recipients and developed a population pharmacokinetics model by considering various factors that might account for alterations in vancomycin pharmacokinetics. Our analyses revealed that lower serum creatinine levels and a shorter duration from the day of LT were associated with higher vancomycin clearance and led to subtherapeutic drug exposure. We also performed Monte Carlo simulations to determine the appropriate dosing strategy in pediatric LT recipients, which revealed that a standard vancomycin dosing might be insufficient and that higher or more frequent dosing might be necessary to achieve an AUC_24_/MIC of ≥400 in pediatric LT recipients with normal renal function. To the best of our knowledge, this is the first study to assess vancomycin pharmacokinetics in pediatric LT recipients by population pharmacokinetics analysis.

## INTRODUCTION

Bacterial infections are a major cause of morbidity and mortality following liver transplantation (LT) (1). Methicillin-resistant Staphylococcus aureus (MRSA) infections, which are increasingly common after LT, include bloodstream infection, surgical site infections, and pneumonia (2). The efficacy of vancomycin, a glycopeptide that is the most commonly used antibiotic for the treatment of severe MRSA infections, is related to its 24-h area under the concentration-time curve (AUC_24_)/MIC ratio. In addition, insufficient vancomycin exposure is associated with increased mortality (3). A trough vancomycin concentration of ≥10 μg/ml has been used as a pharmacodynamic target and surrogate marker of AUC for several decades (4). However, a recently published guideline for therapeutic drug monitoring of vancomycin for serious MRSA infections in adult and pediatric patients recommends the use of AUC as a vancomycin pharmacodynamic target and that an AUC_24_/MIC ratio of 400 to 600 should be achieved in patients with suspected or definitive MRSA infections (5). Given the potential severity of MRSA infections after pediatric LT, achieving appropriate vancomycin exposure is critical in this patient population.

Vancomycin is eliminated primarily by renal filtration (6), and vancomycin clearance is strongly correlated with renal function. In the neonatal and early infantile periods, glomerular filtration rate per body surface area increases rapidly, typically reaching adult levels around 1 year of age (7). In addition, several factors, such as hemodynamic instability due to surgery, sepsis, and nephrotoxicity from calcineurin inhibitors that are commonly used after LT as immunosuppressants, may change renal function after LT (8). In addition, fluid replacement during surgery has also been reported to increase the volume of distribution (*V*) of antibiotics in LT recipients (9). Age and transplant surgery-related physiological changes may also alter vancomycin pharmacokinetics in pediatric LT recipients, resulting in different dosing requirements to maintain drug exposure for the effective treatment of severe MRSA infections while limiting toxicity.

The primary objective of this single-center, retrospective pharmacokinetics study was to characterize the pharmacokinetics of intravenous vancomycin after LT in pediatric patients and to determine appropriate dosing in this patient population.

## RESULTS

### Patient characteristics.

The patient characteristics are shown in Table 1. Briefly, 1,158 serum vancomycin concentrations were determined in 161 subjects, with a total of 270 episodes, during the study period. The median patient age was 13.3 months. The common underlying diseases requiring LT were biliary atresia (*n* = 84, 52%), metabolic diseases (*n* = 30, 19%), and fulminant hepatitis (*n* = 26, 16%). The median sCr was 0.16 mg/dl (interquartile range, 0.12 to 0.23 mg/dl) and ranged between 0.06 and 5.43 mg/dl. The median days from liver transplantation (DFLT), defined as the number of days from the date of LT to the first day of vancomycin treatment, was 17 days, and the majority of the patients received vancomycin within the first month after LT.

**TABLE 1:** Baseline characteristics^*a*^

Characteristic	Median (IQR)	Range
No. of patients	161	
Sex, male/female	72 (44.7%)/89 (55.3%)	
Underlying diseases		
Biliary atresia/metabolic diseases	84 (52.2%)/30 (18.6%)	
Fulminant hepatitis/liver cirrhosis	26 (16.1%)/7 (4.3%)	
Liver fibrosis/vascular abnormalities	7 (4.3%)/4 (2.5%)	
Liver tumor	3 (1.9%)	
Vancomycin treatment episode	270	
Patient age, mo (*n* = 270)	13.3 (7.6–53.5)	1–186
wt, kg (*n* = 270)	9.1 (6.8–16.2)	3.1–61.0
ht, cm (*n* = 270)	69.7 (63.3–99.7)	48.0–175.5
Serum albumin level, g/dl (*n* = 270)	3.1 (2.8–3.3)	1.5–4.8
sCr, mg/dl (*n* = 270)	0.16 (0.12–0.23)	0.06–5.43
Serum ALT (U/liter) (*n* = 270)	54.7 (27.4–134.9)	2.5–1,215
DFLT (*n* = 270)	17 (6–31)	0–357
Vancomycin dose, mg/kg/dose (*n* = 270)	15.0 (14.0–15.0)	4.3–37.0
No. of vancomycin concn	1,158	
Vancomycin concn (*n* = 1,158)	11.9 (7.7–16.0)	0.3–61.6
Tacrolimus level, μg/ml (*n* = 270)	8.2 (5.5–10.4)	1.2–22.6

a ALT, alanine transaminase; CLCr, creatinine clearance; IQR, interquartile range; sCR, serum creatinine.

### Population pharmacokinetics model building.

A one-compartment model was selected as the structural model. An exponential variability error model was used to describe interindividual variability, and an additive/proportional error model was selected to explain residual variability.

DFLT and sCr significantly influenced vancomycin clearance, reducing the objective function value by 435.09 and 21.38, respectively, whereas age, sex, underlying disease, albumin, and alanine aminotransferase did not have an effect. No covariates except for body weight influenced vancomycin volume of distribution (*V*). The final model included the effects of sCr and DFLT on clearance (Table 2). The final pharmacokinetics model (Table 3) was as follows:

$$\text{TVCL}\left(\text{l}/\text{h}\right)={\text{CL}}_{\text{pop}}\times {\left(\text{sCr}/0.\text{16}\right)}^{\theta \text{sCr}}\times {\left(\text{DFLT}/\text{17}\right)}^{\theta \text{DFLT}}\times {\text{wt}}^{0.\text{75}}\times \text{exp}\left(\eta_{\mathrm{CL}}\right)$$

$$\text{TVV}i=V_{\text{pop}}\times \text{wt}\times \text{exp}\left(\eta_{V}\right)$$

where TVCL is typical value of clearance, CL is clearance, sCr is in mg/dl, wt is weight, exp is exposure, *η* is shrinkage, TVV*i* is typical value of *V* in liters, and *V*_pop_ is volume of distribution of the population.

**TABLE 2:** Impact of each covariate^*a*^^,^^*b*^

Analysis type	Covariates added	ΔObjective function
Univariate screening		
Covariates for CL	Age	−0.34
	sCr	−435.09
	Albumin	+2.49
	DFLT	−21.38
	ALT	+3.82
	Sex	+11.65
	Underlying diseases	−18.86
Covariates for *V*	Age	+2.64
	sCr	−5.45
	Albumin	+1.36
	DFLT	−7.58
	ALT	+1.90
	Sex	+11.95
	Underlying diseases	−0.71
Stepwise step for multivariate analysis		
Step 1, sCr for CL	UD for CL	+41.92
	DFLT for CL	−61.27
	DFLT for *V*	−17.94
Step 2, sCr and DFLT for CL	DFLT for *V*	−1.29
Deletion step for multivariate analysis		
sCr and DFLT for CL	sCr for CL	+471.98
	DFLT for CL	+44.62

a ALT, alanine aminotransferase; CL, clearance; DFLT, days from liver transplantation; sCr, serum creatinine; *V*, volume of distribution; UD, underlying disease.

b Sex and underlying disease were used as categorical covariates. For underlying disease, biliary atresia was set as a value of 1, and other disorders were set as a value of 0.

**TABLE 3:** Population pharmacokinetic estimates for the final model and bootstrap results^*a*^

Parameter	Base model estimate (RSE%)	Final model estimate (RSE%)	Median bootstrap value of the final model (95% CI)
Population parameters			
CL (l/kg^0.75^/h)^*b*^			
θ_CL_	0.27 (4.64)	0.29 (4.13)	0.29 (0.26 to 0.32)
θ_DFL_		−0.09 (34.8)	−0.09 (−0.15 to −0.03)
θ_SCr_		−0.70 (8.23)	−0.69 (−0.79 to −0.57)
*V* (l/kg)^*c*^			
θ*_V_*	1.23 (7.56)	1.00 (5.97)	1.00 (0.89 to 1.14)
Between-subject variability			
% ωCL	46.47 (2.88)		
% ωVc	48.14 (6.55)		
Residual variability			
Proportional (%)	56.5 (4.02)		

a CL, total body clearance; DFLT, days from liver transplantation; RSE, relative standard error; sCR, serum creatinine; *V*, volume of distribution; ω, between-subject variability; WT, body weight.

b CL (l/h) = θ_CL_ × wt^0.75^ × (SCr/0.16)^θsCr^ × (DFLT/17)^θDFLT^ × exp(*η*_CL_).

c *V* (l) = θ*_V_* × wt × exp(*η*_CL_).

**FIG 1:**
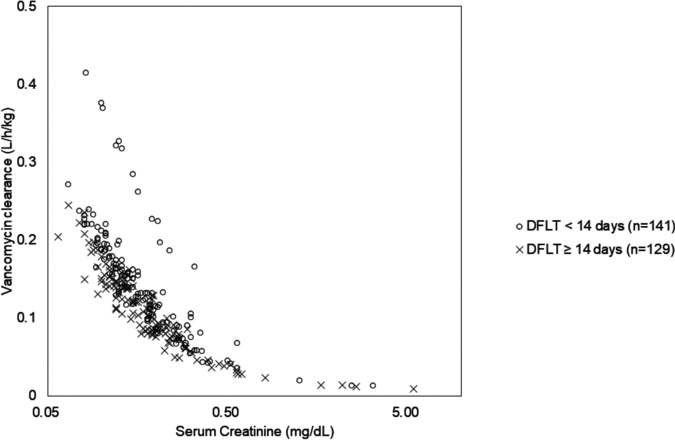
Relationship between serum creatinine and vancomycin clearance between patients with a DFLT of <14 days and those with a DFLT of ≥14 days. *Post hoc* clearance estimates based on serum creatinine and DFLT are shown. Circles and crosses indicate data of patients with a DFLT of <14 days and those with a DFLT of ≥14 days, respectively. DFLT, days from liver transplantation.

In this model, *ηi* was normally distributed with a mean of 0 and a variance of ω^2^, where ω is between-subject variability. The magnitude of ε shrinkage was 4.55%. The model parameters had moderate levels of *η* shrinkage for clearance (22.0%) and *V* (27.0%). The *post hoc* estimation revealed that the average clearance and *V* were 0.18 ± 0.08 liters/h/kg and 1.01 ± 0.35 liters/kg, respectively. We also evaluated the relationship between sCr and vancomycin clearance in patients with DFLT of <14 days and ≥14 days (Fig. 1).

### Model validation.

**FIG 2:**
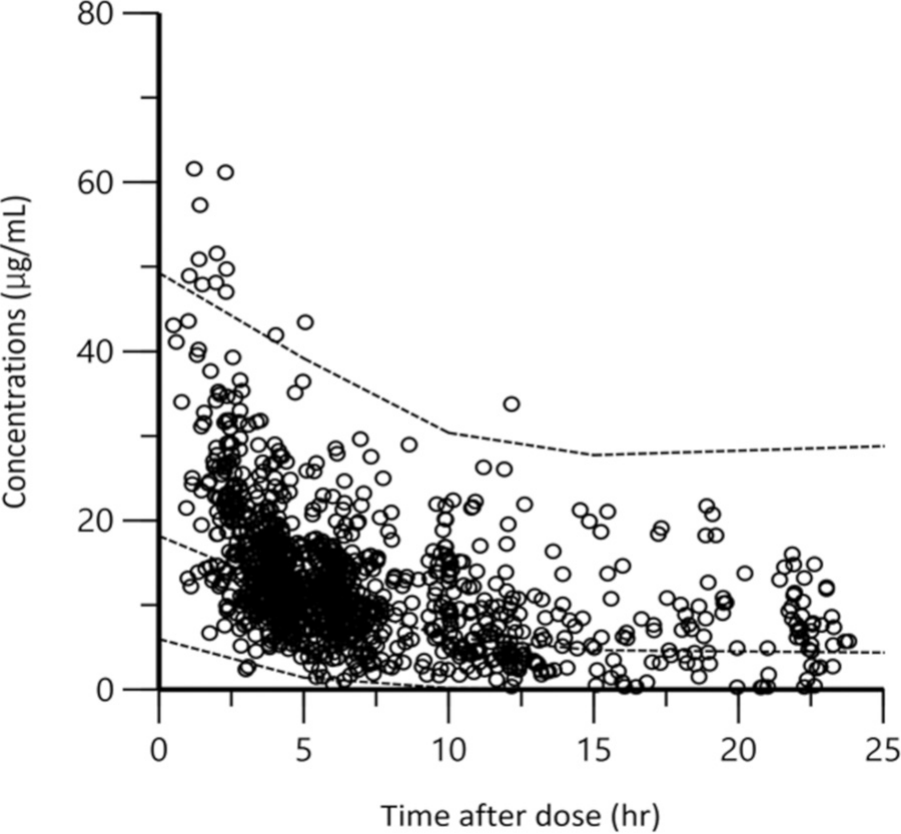
Visual prediction checks of observed and simulated concentrations. Visual predictive checks (*n* = 1,000) of observed vancomycin concentrations (μg/ml) overlaid on medians with 90% prediction intervals of simulated concentrations generated from the final model. The dotted lines are medians with 90% prediction intervals of simulated concentrations.

Table 3 shows the statistical distributions of parameter estimates based on bootstrap analysis. The final model converged on 1,000 bootstrap samples, representing a convergence rate of 100%. The median values of the parameters estimated from the bootstrap analysis were in good agreement, and the 95% confidence intervals were narrow, demonstrating satisfactory precision. The basic goodness-of-fit plots of the final model demonstrated that the scatterplots of the observed concentrations versus population-predicted concentrations and those of the observed concentrations versus individual predicted concentrations were uniformly distributed around the line of identity (Fig. S1A and B). Additionally, the conditional weighted residuals were distributed symmetrically around zero across all population-predicted concentrations and the time after dose range (Fig. S1C and D). The predictive performance of the model observed by the visual prediction check (Fig. 2) showed that the observed concentrations were almost within the 90% predictive intervals of the simulated concentrations.

### Dosing simulations.

Monte Carlo simulations (MCSs) were performed using the final model to determine appropriate dosing regimens based on DFLT and sCr. The MCS results after three dosing regimens (15 mg/kg every 8 h [q8h], 15 mg/kg every 6 h [q6h], and 20 mg/kg q6h) are shown in Table 4. Only 12.1% of the patients with a DFLT of <14 days achieved a trough vancomycin concentration of >10 μg/ml with the 15 mg/kg q6h regimen, in contrast to 82.1% of patients with a DFLT of ≥14 days who achieved the same trough vancomycin concentration. Regarding AUC_24_/MIC, the achievement rate depended on the MIC value. Each regimen showed 100% achievement rate for an MIC of 0.5 μg/ml; however, only 16% of the patients with a DFLT of <14 days achieved an AUC_24_/MIC of ≥400 for an MIC of 1 μg/ml with the vancomycin regimen of 15 mg/kg q6h.

**TABLE 4:** Achievement rate for each vancomycin dosing and days from liver transplantation^*a*^

Vancomycin dosing	Total	DFLT < 14 days	DFLT ≥ 14 days
15 mg/kg every 8 h			
Trough > 10 μg/ml	31.2%	5.2%	58.6%
AUC_24_/MIC > 400 (MIC = 0.5)	100.0%	100.0%	100.0%
AUC_24_/MIC > 400 (MIC = 1)	7.4%	1.3%	9.2%
15 mg/kg every 6 h			
Trough > 10 μg/ml	54.0%	12.1%	82.1%
AUC_24_/MIC > 400 (MIC = 0.5)	100.0%	100.0%	100.0%
AUC_24_/MIC > 400 (MIC = 1)	16.3%	3.3%	29.3%
20 mg/kg every 6 h			
Trough > 10 μg/ml	71.6%	76.3%	100.0%
AUC_24_/MIC > 400 (MIC = 0.5)	100.0%	100.0%	100.0%
AUC_24_/MIC > 400 (MIC = 1)	88.1%	85.6%	100.0%

a AUC, area under the curve; DFLT, days from liver transplantation.

The percentages of patients achieving a vancomycin trough concentration of ≥10 μg/ml or an AUC_24_/MIC of ≥400 according to the dosing regimen (15 mg/kg q8h, 15 mg/kg q6h, 20 mg/kg q6h, or 25 mg/kg q6h) and creatinine clearance (CLCr), as determined by Monte Carlo simulation, are shown in Table S1.

## DISCUSSION

In this first study assessing vancomycin pharmacokinetics in pediatric LT recipients using population pharmacokinetics analysis, we report two major findings. First, in addition to sCr, DFLT was a significant covariate of vancomycin clearance. Second, standard vancomycin dosing may not be sufficient to achieve an AUC_24_/MIC of ≥400 in pediatric LT recipients with normal renal function.

Several studies have previously reported large variations in vancomycin pharmacokinetics in specific pediatric patient populations. Zhao et al. showed that vancomycin clearance in children with hematologic malignancies (0.22 liters/kg/h) was approximately two times higher than that in the general pediatric population (0.103 to 0.114 liters/kg/h) (10). The reported *V* in general pediatric populations ranges from 0.43 and 0.63 liters/kg (11–13), whereas *V* values in specific pediatric populations remain unclear. Our analyses revealed that the clearance was comparable between the pediatric LT recipients and the general population; however, the *V* in these patients was larger than that reported in previous studies. The *V* of vancomycin, a water-soluble agent, is influenced by the amount of water in different body compartments. Pediatric LT recipients usually receive large amounts of fluid in the acute phase after LT to maintain blood flow to the graft, which can cause changes in water storage in the body. In fact, higher *V* values of other water-soluble antibiotics, such as cefotaxime, which have been reported in LT recipients were attributed to large amounts of fluid replacement during surgery (9). In addition, LT recipients occasionally experience ascites due to graft rejection or other causes (14). A larger *V* in pediatric LT recipients might be partially explained by these factors.

We found that DFLT was a significant covariate for vancomycin clearance. Time-dependent changes in the pharmacokinetics of hepatically metabolized drugs, such as tacrolimus, have been well described after LT (15, 16). However, time-dependent changes in the pharmacokinetics of water-soluble, renally eliminated drugs, including antibiotics, have rarely been investigated. In the present study, we found that shorter time after LT was associated with higher vancomycin clearance. Inflammation is a well-known factor that leads to increased clearance of renally eliminated antibiotics through a mechanism termed augmented renal clearance, which has been reported to occur in the presence of vancomycin (17). LT is directly associated with inflammation, and infectious complications, which often occur during the acute phase after LT, are also a source of inflammation (18–20). Although there is no direct evidence, these factors might have also contributed to the higher vancomycin clearance observed in the acute phase after LT in the current study.

The usefulness of AUC as a predictor of vancomycin efficacy is debated (21, 22), yet a recently published consensus guideline recommends AUC as a marker related to vancomycin efficacy (5). In general, an AUC_24_/MIC of ≥400 is the preferred pharmacodynamic target to achieve optimal therapeutic response in adult patients. Evidence on the appropriate AUC_24_/MIC target for pediatric patients remains limited; however, achieving an AUC_24_/MIC of ≥400 is also recommended in the pediatric population (5). The current study results revealed that standard vancomycin dosing was not sufficient in achieving an AUC_24_/MIC of ≥400 in pediatric LT recipients with the assumed MIC of 1 μg/ml, suggesting that higher dosing may be needed. Similar results have been reported in other specific pediatric populations. For example, Hadi et al. performed MCS using population parameters derived from pediatric cancer patients and demonstrated that a vancomycin dose of 60 mg/kg/day achieved an AUC_24_/MIC of ≥400 and a trough vancomycin concentration of ≥15 μg/ml in only 21.5% and 11% of virtual subjects, respectively (23). Moreover, Zhao et al. reported that only 15% to 24% of pediatric patients with hematological malignancies achieved an AUC_24_/MIC of ≥400 with the standard vancomycin regimen of 60 mg/kg/day regimen and that 80 to 90 mg/kg/day vancomycin was required to achieve this target in about 50% of the patients (10). These findings suggest that improvements in individualized vancomycin dosing recommendations should be considered in specific pediatric populations and that therapeutic drug monitoring is essential in these circumstances.

In the present study, we also explored optimal vancomycin dosing in specific situations. With a vancomycin MIC of 1 μg/ml for patients with a CLCr of 21 to 40 ml/min/1.73 m^2^, 15 mg/kg q8h vancomycin was appropriate regardless of the DFLT. In patients with a CLCr of 41 to 60 ml/min/1.73 m^2^, 15 mg/kg q6h vancomycin might be needed within 14 DFLT. After 14 DFLT, vancomycin dosing might be reduced to 15 mg/kg q8h. In patients with a CLCr of 60 to 90 ml/min/1.73 m^2^, 15 to 20 mg/kg q6h vancomycin might be necessary to achieve an AUC_24_/MIC of ≥400. In patients with a CLCr of >90 ml/min/1.73 m^2^, 20 mg/kg q6h vancomycin might be necessary to achieve an AUC_24_/MIC of ≥400. With a vancomycin MIC of 2 μg/ml, the target AUC_24_/MIC might not be achievable by any of these regimens in patients with a DFLT of <14 days and normal renal function. Notably, the robustness of our conclusions is decreased by few patients with severe renal impairment included in the study and lack of information about other nephrotoxic medications (in addition to tacrolimus).

This study has several limitations. First, this was a single-center study in a relatively homogenous patient population, and the generalizability of the findings might be limited. The median age (interquartile range) of the study population was 13.3 (7.6 to 53.5) months, and no neonates and only a few adolescents were included. Therefore, our results should not be broadly extrapolated to neonates and adolescents. Second, we could not assess the clinical efficacy or adverse events related to the AUC_24_/MIC or trough levels of vancomycin. Lastly, we only explored the appropriateness of conventional intermittent intravenous infusion regimens. Intermittent infusion is a standard vancomycin administration method; however, administration via continuous infusion has been suggested as an alternative for patients in whom the AUC target cannot be achieved (5).

In conclusion, usual vancomycin dosing might not be sufficient to achieve an AUC_24_/MIC of ≥400 in pediatric LT recipients with normal renal function. Further pharmacokinetic/pharmacodynamic studies are needed to evaluate the relationship of AUC_24_/MIC with efficacy and toxicity of vancomycin to determine optimal dosing strategies in this patient population.

## MATERIALS AND METHODS

This single-center, retrospective pharmacokinetics study was conducted at the National Center for Child Health and Development (NCCHD), a tertiary children’s hospital in Tokyo, Japan. The NCCHD has the largest pediatric LT center in Japan and performs approximately 60 pediatric transplants annually. Clinical data from pediatric LT recipients under 18 years of age with suspected or proven infection who received intravenous vancomycin between 2006 to 2014 were collected. Data on vancomycin dosing history, serum vancomycin concentrations, age, sex, weight, height, underlying diseases, and the levels of serum albumin, sCr, alanine aminotransferase, and tacrolimus were extracted from the electrical medical records. Data within 14 days from the initiation of vancomycin were analyzed. Patients who received renal replacement therapy were excluded from the study.

Ethical approval to conduct the present study was obtained from the Ethics Committee in the NCCHD (NCCHD-1187).

### Vancomycin dosing and therapeutic drug monitoring strategy.

In the study institution, vancomycin in patients without renal dysfunction was generally initiated with a dosing of 15 mg/kg q6h in patients between the ages of 1 month and 12 years and a dosing of 15 mg/kg q8h in patients between the ages of 13 and 17 years; the dosing was adjusted according to therapeutic drug monitoring results based on trough concentrations, which were generally measured immediately before the fourth or fifth vancomycin dose. The target trough vancomycin level, which was 10 to 15 μg/ml in most situations, was occasionally set to 15 to 20 μg/ml in critically ill patients. AUC-guided therapeutic drug monitoring was not introduced during the study period.

### Measurement of vancomycin concentrations and pharmacokinetics analysis.

Serum vancomycin concentrations were measured in the clinical laboratory of the study institution using a validated fluorescence polarization immunoassay (AxSYM Vancomycin II; Abbott Japan, Tokyo, Japan) between 2006 and 2010 and by a chemiluminescent immunoassay (Architect i Vancomycin^ST^; Abbott Japan) between 2010 and 2014. The correlation of the two assays was confirmed using 192 samples in the range of 2.1 to 94.3 μg/ml; an acceptable good correlation was shown with a correlation coefficient of 0.996, a slope of 0.925, and an intercept of 0.05. The average bias was −4.41% (95% confidence interval, −19.51% to 10.70%) within the typical therapeutic range (5 to 40 μg/ml) according to the Architect i Vancomycin^ST^ package insert (24).

Population pharmacokinetics parameters were determined using the Phoenix NLME 8.2 software (Certara USA, Princeton, NJ, USA) with first-order conditional estimation. One- and two-compartment models were tested. Interindividual variability in structural model parameters was evaluated using an exponential variability error model. Additive, proportional, exponential, and additive/proportional error models were tested in every model to explain residual variability. The TVCL was scaled allometrically by subject weight (weight^0.75^), and typical value of the *V* (TVV) was also scaled by subject weight (weight^1.0^) before evaluation of other covariates (25). In covariate analysis, the influences of age, sex, underlying disease, sCr, albumin, alanine aminotransferase, and DFLT on vancomycin pharmacokinetics were investigated by stepwise forward inclusion and backward elimination, with *P* values of <0.05 and <0.005, respectively. Empirical Bayesian estimates of individual patient pharmacokinetics parameters were generated from the final model using the POSTHOC subroutine. Appropriateness of the final model was confirmed by bootstrap validation (*n* = 1,000) and visual prediction check (VPC). MCSs were performed using the final population pharmacokinetics model to determine appropriate vancomycin doses and intervals for pediatric LT recipients.

The demographic information on patients was obtained from the patient data set. Various dosing intervals were tested. The primary pharmacokinetic/pharmacodynamic target for vancomycin was trough concentration or AUC_24_/MIC. In the present study, a trough vancomycin concentration of ≥10 μg/ml or an AUC_24_/MIC of ≥400 was used to represent an appropriate target to achieve sufficient therapeutic effect. The MCSs of the population data were performed using Microsoft Excel to reflect the expected range of variability of response under the final model assumptions. A total of 1,000 concentration-time profiles were simulated using the parameter estimates obtained from the final model. The Schwartz formula was used to estimate CLCr (26).
