# Supplementary material for: Population Pharmacokinetics and Dosing Optimization of Vancomycin in Pediatric Liver Transplant Recipients
Source: Microbiol Spectr. 2021 Oct 6;9(2):e00460-21. doi: 10.1128/Spectrum.00460-21 (PMC8510181; doi:10.1128/Spectrum.00460-21)
Supplement: SUPPLEMENTAL FILE 1 — Supplemental material. Download SPECTRUM00460-21_Supp_1_seq2.pdf, PDF file, 0.2 MB [file spectrum00460-21_supp_1_seq2.pdf]

Figure S1. Goodness of fit plots for the final population pharmacokinetic model.

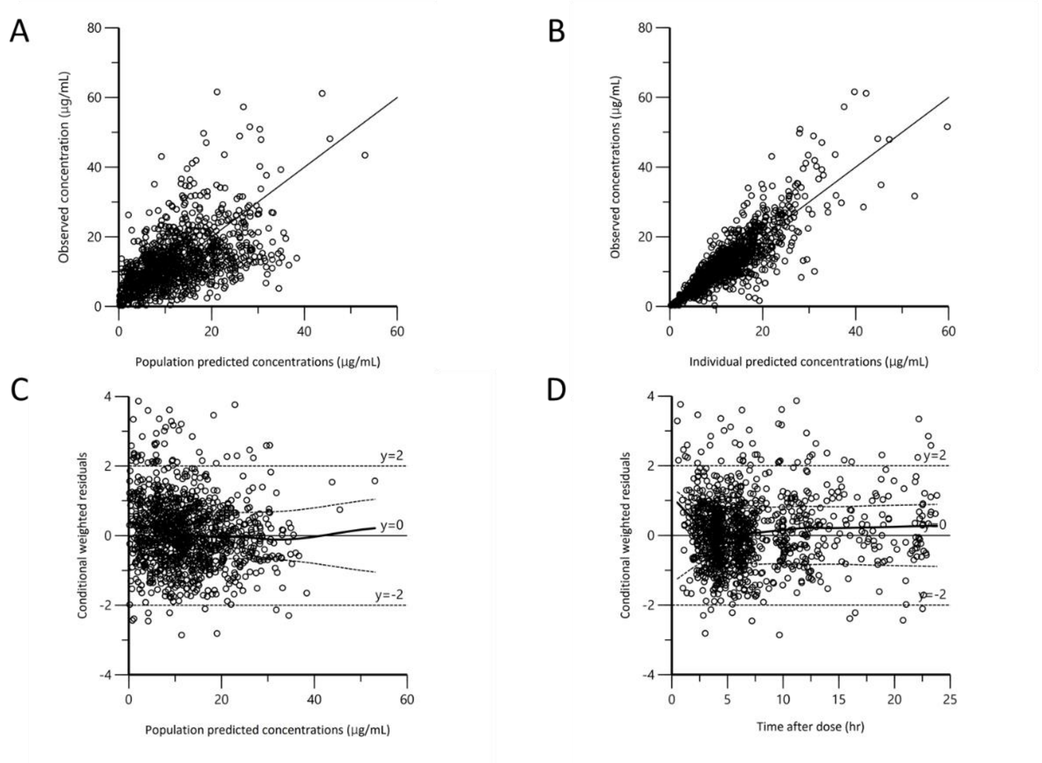

(A) Plot of observed vancomycin concentrations (µg/ml) versus population predicted concentrations (µg/ml). (B) Plot of observed vancomycin concentrations (µg/ml) versus individual predicted concentrations (µg/ml). (C) Plot of conditional weighted residuals versus population predicted concentrations. (D) Plot of conditional weighted residuals versus time after the last dose. (A, B) The solid lines are

lines of unity (y = x). (C, D) Upper dotted curves are locally weighted scatterplot smoothing fitted to the absolute values of the residuals. Solid middle lines are locally weighted scatterplot smoothing fitted to the raw residuals.

Table S1. Monte Carlo simulations for dosing optimization based on renal function in pediatric liver transplant recipients

CLCR (mL/min/1.73 m^2)	Dose	Days from LT, 0–14				Days from LT, 15–30			
		AUC_{24}/MIC > 400			Trough > 10 µg/mL	AUC_{24}/MIC > 400			Trough > 10 µg/mL
		0.50	1.00	2.00		0.50	1.00	2.00	
21–40	15 mg/kg/dose q8h	100.0	100.0	57.3	100.0	100.0	100.0	99.8	100.0
	15 mg/kg/dose q6h	100.0	100.0	100.0	100.0	100.0	100.0	100.0	100.0
	20 mg/kg/dose q6h	100.0	100.0	100.0	100.0	100.0	100.0	100.0	100.0
	25 mg/kg/dose q6h	100.0	100.0	100.0	100.0	100.0	100.0	100.0	100.0
41–60	15 mg/kg/dose q8h	100.0	99.8	0.0	100.0	100.0	100.0	0.0	100.0
	15 mg/kg/dose q6h	100.0	100.0	29.6	100.0	100.0	100.0	92.1	100.0
	20 mg/kg/dose q6h	100.0	100.0	97.1	100.0	100.0	100.0	100.0	100.0
	25 mg/kg/dose q6h	100.0	100.0	100.0	100.0	100.0	100.0	100.0	100.0
60–90	15 mg/kg/dose q8h	100.0	42.8	0.0	100.0	100.0	88.6	0.0	100.0
	15 mg/kg/dose q6h	100.0	75.3	0.9	100.0	100.0	100.0	24.9	100.0
	20 mg/kg/dose q6h	100.0	100.0	64.7	100.0	100.0	100.0	95.6	100.0

	25 mg/kg/dose q6h	100.0	100.0	100.0	100.0	100.0	100.0	100.0	100.0
>90	15 mg/kg/dose q8h	100.0	3.5	0.0	32.5	100.0	15.6	0.0	69.8
	15 mg/kg/dose q6h	100.0	26.2	0.0	69.6	100.0	46.5	0.0	100.0
	20 mg/kg/dose q6h	100.0	90.8	0.0	99.5	100.0	100.0	0.9	100.0
	25 mg/kg/dose q6h	100.0	100.0	42.0	100.0	100.0	100.0	97.3	100.0

CLCR, creatinine clearance; LT, liver transplantation; AUC_{24}, 24-h area under the concentration-time curve; MIC, minimum inhibitory concentration
